# Progress in the Research and Targeted Therapy of ErbB/HER Receptors in Urothelial Bladder Cancer

**DOI:** 10.3389/fmolb.2021.800945

**Published:** 2021-12-23

**Authors:** Dong Chen, Yunlin Ye, Shengjie Guo, Kao Yao

**Affiliations:** Department of Urology, State Key Laboratory of Oncology in South China, Collaborative Innovation Center for Cancer Medicine, Sun Yat-sen University Cancer Center, Guangzhou, China

**Keywords:** urothelial carcinoma, ErbB (EGFR), (HER), bladder cancer (BCa), urothelial carcinoma (UC)

## Abstract

Bladder cancer is a lethal malignancy and a majority of bladder cancer arise from urothelial cells. Infiltration and metastasis are barriers for the radical cystectomy to achieve favored outcome and are the main cause of death. Systemic therapy, including chemotherapy, targeted therapy, and immunotherapy, is fundamental for these patients. erbB/HER receptors are found to be overexpressed in a subgroup of urothelial carcinoma, targeting erbB/HER receptors in these patients was found to be an efficient way in the era of genetic testing. To evaluate the role of erbB/HER receptors in bladder cancer, we reviewed the literature and ongoing clinical trials as regards to this topic to unveil the context of erbB/HER receptors in bladder cancer, which probably help to solidate the theoretical basis and might instruct further research.

## Introduction

Globally, there were an estimated 573,278 new cases of bladder cancer in 2020 and causes 212,536 deaths. Bladder cancer has been ranked the 10th frequently diagnosed cancer and more men were diagnosed than women in all region included (Sung et al., 2021). A majority of urinary bladder cancer that arise from translational epithelium are termed urothelial carcinoma, the other types of bladder cancer, including squamous cell carcinoma, adenocarcinoma, small cell carcinoma, and sarcoma, only account for a small proportion (<5%) in together and usually apply to a different therapy principle from urothelial carcinoma. Among all urothelial bladder carcinoma, 80% of patients are non-muscle invasive, routine transurethral resection of tumor followed by a single dose of intravesical chemotherapy or intravesical BCG is sufficient for these patients, yet around 20% of them are muscle invasive at the time of diagnose, which will probably lead to metastasis or recurrence after a radical surgical resection, dramatically decrease 5-year survival rate from 82–100–11.6% (Slojewski, 2000). Systemic therapy for locally advanced or metastatic urothelial bladder cancer is essential to improve survival. A platinum-based chemotherapy is still the standard therapy for metastatic urothelial carcinoma but lack long-term effectiveness and tolerance in elderly patients (Galsky et al., 2018). Regiment of chemotherapy and checkpoint PD-1/PD-L1 inhibitors are challenging a better survival in these patients.

Bladder cancer is a highly heterogenic tumor and need a precise therapy individually, thus, it is necessary to comprehensively consider the specific clinical condition, pathological classification, immune and molecular feature of the patient and develop a personalized treatment plan. For localized advanced and metastatic urothelial carcinoma patients, systemic therapy is fundamental to achieve a better survival. Therefore, more and more medications were discovered and used as single agent or combined regiment in the past decades. Along with the development of precision medicine, targeted therapy is coming to the center stage of systemic therapy, finding valuable therapeutic targets is hotspot of the current research. Studies have shown that the abnormal expression of ErbB/HER receptors in bladder cancer is similar as in breast cancer and non-small cell lung cancer (Roskoski, 2014). This study systematically reviewed the relevant literature on ErbB/HER receptors and bladder cancer, to better understand the role of the four ErbB/HER receptors in bladder cancer and its effectiveness as a therapeutic target. It is also expected that the literature review can provide necessary information for further clinical research.

## ErbB/HER Family Members in Bladder Cancer

### ErbB/HER Family Signaling Pathway

The ErbB/HER family of tyrosine kinase receptors comprises four family members: HER1/EGFR/erbB1, HER2/erbB2/neu, HER3/erbB3, and HER4/erbB4 (Lemmon and Schlessinger, 2010). The family members share similar molecular structure including an extracellular domain (ECD) with two cysteine-rich regions, a single transmembrane of membrane-spanning region, a juxta-membrane cytoplasmic domain, and an intracellular kinase with multiple C-terminal tyrosine residues (Yarden and Sliwkowski, 2001; Oda et al., 2005). Heterodimerization of ErbB/HER family receptors induces the autophosphorylation of tyrosine residues within the cytoplasmic domain of the heterodimer and trigger a complex pathway system leading to cellular proliferation and tumorigenesis (Jin and Esteva, 2008). Many studies have unveiled the mechanism of EGFR receptors/ligand interaction in the tumor origination and progression (Normanno et al., 2006; Galsky and Hall, 2010). Three main signaling pathways are found to be the downstream of EGFR: the Ras/Raf/MAPK pathway, the PI3K/AKT/mTOR pathway, and the STAT pathway (Jorissen et al., 2003; Su et al., 2007), aberrant activation of these pathways responds to tumorigenesis in several solid tumors including bladder cancer.

### ErbB/HER Family Members in Bladder Cancer

ErbB1/HER1, known as EGFR, was discovered as an oncogenic target in NSCLC, glioblastoma, and basal-like breast cancers. Overexpression of HER1 and its relevance to bladder cancer was at first reported in 1997 by Liukkonen et al. (1997), Liukkonen et al. (1999). HER1 was found positive in 75% of primary bladder cancer and relevant metastases were positive in 86% of the HER1-positive tumor cases (Carlsson et al., 2015). Protein shedding of HER2 in urine was reported to be a prognostic biomarker for urothelial bladder carcinoma (Bryan et al., 2015). Moreover, HER1 expression of cancer cell prompt disease progression in muscle invasive bladder cancer patients after chemotherapy (Kim et al., 2014; Daizumoto et al., 2018; Wang et al., 2020).

HER2, receptor tyrosine-protein kinase erbB-2, is a member of the ErbB/HER family of receptor tyrosine kinases. Overexpression of HER2 results in the autophosphorylation of tyrosine residues within the cytoplasmic domain of the heterodimer and initiates a variety of signaling pathways leading to proliferation and tumorigenesis (Yarden and Sliwkowski, 2001). HER2 is an established prognostic and therapeutic target for a variety of malignances such as breast cancer, gastric cancer et al (Krüger et al., 2002). Unlike breast cancer, where the role of HER2-targeting agents has been well established in both metastatic and adjuvant settings for decade, the efficacy of HER2-targeting agents in bladder carcinomas still lack large-scale clinical research. Due to the relevance of appropriate patient selection to therapy efficacy, the accurate assessment of HER2 status is fundamental. In a multicenter randomized phase II trial, addition of trastuzumab to platinum-based chemotherapy with gemcitabine show no better response and survival in unselected local-advanced and metastatic bladder cancer population (Oudard et al., 2015). Accordingly, HER2-targeted therapies need to be tested in patients with bladder cancer harboring HER2 overexpression, and/or amplifications or other mutations of ERBB2 which encodes HER2. Based on published studies, HER2 overexpression was observed in 9.2–12.4% of invasive bladder carcinoma and 5.1% of that were proven having a *HER2* gene amplification (Laé et al., 2010; Oudard et al., 2015; Yan et al., 2015). Moreover, HER2 alterations were higher in the luminal than in the basal subtypes, higher in metastases than primary tumors (Fleischmann et al., 2011). However, the expression of HER2 in protein level probably represents as hint for effective binding with monoclonal HER2 antibody for targeted therapy (Kiss et al., 2017).

The function of HER3 and HER4 in bladder cancer are still lacking sufficient research up to date. Prognostic value of HER3 and HER4 is preliminary studied in bladder cancer, higher soluble HER3 expression is associated with improved survival in bladder cancer, which may attribute to that sHER3 inhibits cancer cell growth and migration (Memon et al., 2004; Memon et al., 2018). Similar to HER3, bladder cancer tumors expressing specific HER4 isoform demonstrate improved patient survival in the absence of ER-α (Junttila et al., 2003; Memon et al., 2004), further research indicate that ER-α inhibition may be a useful treatment for bladder cancer patients with overexpression of both ER-α and HER4 (Munk et al., 2013). Interestingly, HER4 may rely on an interaction with HER4 to delay the tumor progress, metastasis, and the resistance to trastuzumab therapy. Like the situation in bladder cancer, presence of HER4 expression was also independent predictor of favored outcome in breast cancer and the head and neck squamous cell carcinoma (Barnes et al., 2005; Machleidt et al., 2013; De Pauw et al., 2018). However, HER4 expression correlate with an unfavorable clinical outcome in patients with colorectal cancer (CRC) and gastric cancer (Yun et al., 2018; Jia et al., 2020). The role of HER4 and its isoforms were still to be furtherly explored. The expression of HER3, HER4, may correlate with HER1 and HER2 to impact the signal transduction of HER1/HER2 function (Memon et al., 2006).

## ErbB/HER Targeted Therapy in Bladder Cancer

### Small Molecule EGFR Tyrosine Kinase Inhibitors Therapeutics in Urothelial Cancer

EGFR Tyrosine kinase inhibitors (TKI) bind to the tyrosine kinase domain in the epidermal growth factor receptor and undermine the EGFR function of cancer cell to cure the cancer. There is high risk of drug resistance for TKI, especially first-generation EGFR-TKI (Erlotinib, Gefitinib) when the combination to the target is reversible. The second-generation EGFR-TKI (Afatinib, Dacomitinib) has improved the molecular structure to lower the risk of drug resistance. In addition to reversibly binding to the ATP binding site on EGFR, it can also be alkylated or covalently bonded with EGFR-specific amino acid residues which is an irreversible bond. The new generation of EGFR TKIs, such as Osimertinib, furtherly target in several new mutants which is found in drug-resistance cases to improve the overall survival. Many EGFR TKIs are explored as efficient therapeutics for breast cancer, NSCLC or gastric cancer, however, only a few of them are further investigated in bladder cancer. Emerging studies focus on the precision therapy after genetic screening before deciding suitable targeted therapy, for instance, the MATCH trial (NCT:02465060).

The first generation of TKIs are not broadly studied as surrogate of chemotherapy in advanced urothelial cancer. Only a small number of clinical trials tested the usefulness of Erlotinib as an neoadjuvant- or adjuvant therapy in muscle-invasive bladder cancer, Pruthi et al. (2010), reported that Erlotinib can benefit the surgical pathology and short-term outcomes in patients undergoing radical cystectomy, five of twenty patients with clinical stage T2 disease were found to be pathological complete response after the neoadjuvant Erlotinib therapy. However, further big-scale randomized trials are still needed to maximize oncological outcome and avoid overtreatment. For Gefitinib, few study was executed in bladder cancer patients, limited preclinical research shows that Gefitinib can inhibit cell proliferation of bladder cancer cell (Deng et al., 2019; Rose et al., 2020).

Afatinib and Dacomitinib represent the second generation of EGFR-TKIs. Both are highly selective TKIs that covalently bind to HER1, HER2, and HER4 to irreversibly inhibit tyrosine kinase autophosphorylation and downregulate HER signaling (Modjtahedi et al., 2014). Afatinib and Dacomitinib show anti-tumor effect and synergism with radiation therapy in preclinical research (Grivas et al., 2013; Tsai et al., 2015), nevertheless, few positive results have been achieved in clinical trial.

Lapatinib is a small molecule dual tyrosine kinase inhibitor, targeting both the epidermal growth factor receptor, HER1 and HER2. It can across the blood-brain barrier and can be used in combination with trastuzumab or as a single reagent. Preclinical results found that activation of HER2 predict a greater response to Lapatinib (de Martino et al., 2014). However, the phase III randomized trial comparing Lapatinib maintenance versus placebo after first-line chemotherapy in patients with HER1 or HER2 positive metastatic bladder cancer, Lapatinib arm shows no significant improvements in outcome comparing with the placebo arm, like the results from previous phase II trial (Wülfing et al., 2009; Powles et al., 2017).

### ErbB/HER Receptors-Targeted Monoclonal Antibodies and Antibody-Drug Conjugates (ADC) Therapeutics in Urothelial Cancer

Cetuximab (also known as IMC-C225) is the most advanced clinically advanced anti-EGFR human/rat chimeric monoclonal antibody, which binds to epidermal growth factor receptors (EGFR) with high specificity and has been developed as therapeutic reagent to multiple solid tumors in preclinical trials. It has been shown to be effective for a variety of tumors, including head and neck squamous cell carcinoma, non-small cell lung cancer and colorectal cancer. Cetuximab activity is related to PI3K/AKT and KRAS/RAF/MAPK signaling pathways functionality and its activity has been shown to be higher in wild-type KRAS tumors. However, mutation of KRAS have been reported in 10–15% of urothelial carcinoma (Juanpere et al., 2012), which restrict the application of Cetuximab in bladder cancer as a single reagent (Wong et al., 2012), the regimen including Cetuximab and chemotherapy or immunotherapy may be a promising trying for further trial.

In 2007, a multicenter phase II trial showed that a regimen of Trastuzumab, Paclitaxel, Carboplatin, and Gemcitabine (TPCG), among the 44 HER2-positive patients, 31 patients (70%) got a partial or complete response (Hussain et al., 2007). To determine the true contribution of Trastuzumab in this regimen, Oudard et al. (2015) initiate a multicenter randomized phase II trial of gemcitabine plus platinum with or without Trastuzumab in advanced or metastatic urothelial carcinoma overexpressing HER2, finding that HER2-positive patients account for only 13.3% of 563 screened patients, moreover the comparison showed no significant difference in median mPFS, ORR, and mOS.

Combination of Trastuzumab and chemotherapy was found efficient in recurrent urothelial bladder carcinoma with HER2 GENE amplification in a case report as a second-line regimen, the patient got a clinically complete remission (CR) for 34 months after five cycles of trastuzumab 6 mg/kg every 3 weeks after a loading dose of 8 mg/kg and cisplatin 75 mg/m^2^ every 3 weeks (Jiang et al., 2020). A similar recurrent and metastatic urothelial carcinoma case received Trastuzumab and Gemcitabine as an experimental third-line treatment also acquire a complete tumor remission after 8 cycles of therapy (Wezel et al., 2018). However, the most efficient way to screen out suitable patients for this treatment combination is still unclear. To evaluate the HER2-targeted therapy for recurrent bladder cancer patients with *HER2* amplification (≥7 copy numbers) based on genetic testing, the phase II MATCH trial included recurrent bladder cancer patients that have progressed following at least one line of standard treatment of for which no agreed upon treatment approach exists.

The preclinical and a phase II trial find that these patients can benefit from HER2-targeted Trastuzumab or Trastuzumab-emtansine (T-DM1), an ADC consisting of trastuzumab linked to the tubulin-binding agent DM1 via a stable thioether linker (Hayashi et al., 2015). Except for DM1, another cytotoxic payload monomethyl auristatin E (MMAE) (Sheng et al., 2021) is also established linking to the HER2 antibody. Comparing with other ADCs targeting Nectin-4 and TROP2, which are expecting results from phase III trials, HER2-targeted ADCs (Trastuzumab Emtansine, RC48-ADC) are still in earlier stage of development, but more promising ADCs are emerging and will be competitive as basic therapy for advanced urothelial cancer (Mollica et al., 2020; Sarfaty and Rosenberg, 2020).

Pertuzumab, also a recombinant humanized anti-HER2 IgG1 monoclonal antibody, binds to the extracellular dimerization domain of HER2 and inhibits the ligand induced HER2 heterodimerization. Pertuzumab has a synergistic effect with Trastuzumab in multiple types of cancer. Investigation of Pertuzumab as a therapeutic option in bladder cancer patients with HER2 mutant is still ongoing (NCT02091141).

## Conclusion

Recently, the systemic therapy of urothelial cancer developed rapidly, including chemotherapy, immunotherapy, targeted therapy, antibody-conjugated therapy, and other treatment methods. The novel treatment rapidly entered the first-line metastatic urothelial carcinoma (mUC) candidate list after the success as a second line option, then furtherly entered the exploration of the perioperative treatment of MIBC. Various treatments for mUC have achieved great success. In recent years, the FDA has approved immunotherapy, FGFR inhibitors, and ADC drugs (EV) for the second-line treatment of mUC.

However, except for pembrolizumab, most of these approved second-line treatments lack the overall survival (OS) benefit data of the Phase III study, so they need to be treated with caution in clinical application. The standard first-line treatment of mUC patients is still cisplatin-based chemotherapy. For patients who are intolerant to cisplatin, immunotherapy can be used as the first-line treatment. The combination of chemotherapy and immunotherapy has theoretical advantages, and small-sample studies have confirmed its effectiveness, but there is still a lack of phase III clinical data to support this treatment strategy to achieve better OS. Fortunately, the PD-L1 antibody avelumab was used as a maintenance treatment for patients with effective first-line chemotherapy and reported the benefits of OS. Therefore, the prospect of combination therapy is still relatively broad, but further research is needed to select the appropriate population.

In patients with urothelial carcinoma without metastasis, neoadjuvant therapy has become a hot area of research interest. At present, a series of studies have been carried out on the neoadjuvant treatment of MIBC, and the pathological complete response rate of the neoadjuvant therapy is 31–46% to achieved satisfied tumor control. However, the final purpose of neoadjuvant treatment before radical cystectomy is to prolong OS. The OS data in these studies is not solid for that the OS is influenced by the surgical technique, lymph node dissection range, postoperative adjuvant treatment, and subsequent treatment after metastasis or recurrence. Therefore, the impact of these new treatment strategies on OS needs to be further confirmed by the Phase III study.

The therapy targeting the HER receptors has shown a promising clinical outcome in some small-sample trials, a genetic testing before the targeted therapy is essential for patient selection and the combination of HER-targeted therapy and chemotherapy probably will obtain better survival. HER2-targeted therapy has shown promising result in several trials, the development of HER2 monoclonal antibody and antibody-drug conjugates is emerging and will probably challenge the first-line therapy in patients with positive HER2 expression.

We went through the clinicaltrials.gov and found some early stage trails concerning HER family and bladder cancer (see Table 1). Still, more phase 3/4 trails are ungently needed to make a final conclusion on the role of HER receptors in bladder cancer. According to the new updated data from American Society of Clinical Oncology (ASCO) conference abstracts, more ADC drugs spring up and we can expect better survival outcomes of these drugs.

**TABLE 1:** Results from clinical trials of HER2-targeted antibodies or ADC drugs.

	Line	Phase	Case number	Overall response rate	mPFS	OS	3/4 TRAEs	Trial registration ID	References
**Cetuximab + paclitaxel**	Neoadjuvant	II	28	25%	16.4 weeks	42 weeks	Rash, fatigue, low magnesium	NCT00350025	44
**Trastuzumab + carboplatin + gemcitabine + paclitaxel**	Advanced urothelial carcinoma	II	44	70%	9.3 m	14.1 m	Myelosuppression, sensory neuropathy, cardiac toxicity	NCT00151034	45
**RC48-ADC**	Locally advanced and metastatic urothelial carcinoma	II	43	51.2%	6.9 m	13.9 m	Hypoesthesia, neutropenia	NCT03507166	46

## Abbreviations

ADC, antibody-drug conjugate; Akt Protein kinase B; EGFR, epidermal growth factor receptor; HER, human epidermalgrowth factor receptor; KRAS, Kirsten rate sarcoma viral oncogene; PD-1, Programmed Cell Death 1; PD-L1, Programmed Cell Death Ligand 1; RAF, Rapidly Accelerated Fibrosarcoma; MAPK, Mitogen-activated protein kinase; MIBC, muscle-invasive bladder cancer; MMAE, monomethyl auristatin E; mTOR, Mechanistic target of rapamycin; NSCLC, non-small cell lung cancer; ORR, objective response rate; OS, overall survival; PI3K, Phosphoinositide 3-kinase; PFS, Progression-free survival; TKI, Tyrosine kinase inhibitor; T-DM1, Trastuzumab-emtansine; UC, urothelial cancer.

## References

[B1] Barnes N. L. P. Khavari S. Boland G. P. Cramer A. Knox W. F. Bundred N. J. (2005). Absence of HER4 Expression Predicts Recurrence of Ductal Carcinoma *In Situ* of the Breast. Clin. Cancer Res. 11 (6), 2163–2168. 10.1158/1078-0432.ccr-04-1633 15788662

[B2] Bryan R. T. Regan H. L. Pirrie S. J. Devall A. J. Cheng K. K. Zeegers M. P. (2015). Protein Shedding in Urothelial Bladder Cancer: Prognostic Implications of Soluble Urinary EGFR and EpCAM. Br. J. Cancer 112 (6), 1052–1058. 10.1038/bjc.2015.21 25719831 PMC4366887

[B3] Carlsson J. Wester K. De La Torre M. Malmström P.-U. Gårdmark T. (2015). EGFR-expression in Primary Urinary Bladder Cancer and Corresponding Metastases and the Relation to HER2-Expression. On the Possibility to Target These Receptors with Radionuclides. Radiol. Oncol. 49 (1), 50–58. 10.2478/raon-2014-0015 25810701 PMC4362606

[B4] Daizumoto K. Yoshimaru T. Matsushita Y. Fukawa T. Uehara H. Ono M. (2018). A DDX31/Mutant-P53/EGFR Axis Promotes Multistep Progression of Muscle-Invasive Bladder Cancer. Cancer Res. 78 (9), 2233–2247. 10.1158/0008-5472.can-17-2528 29440146

[B5] de Martino M. Zhuang D. Klatte T. Rieken M. Rouprêt M. Xylinas E. (2014). Impact ofERBB2mutations on *In Vitro* Sensitivity of Bladder Cancer to Lapatinib. Cancer Biol. Ther. 15 (9), 1239–1247. 10.4161/cbt.29687 24971884 PMC4128866

[B6] De Pauw I. Lardon F. Van den Bossche J. Baysal H. Fransen E. Deschoolmeester V. (2018). Simultaneous Targeting of EGFR , HER 2, and HER 4 by Afatinib Overcomes Intrinsic and Acquired Cetuximab Resistance in Head and Neck Squamous Cell Carcinoma Cell Lines. Mol. Oncol. 12 (6), 830–854. 10.1002/1878-0261.12197 29603584 PMC5983215

[B7] Deng J. Peng M. Wang Z. Zhou S. Xiao D. Deng J. (2019). Novel Application of Metformin Combined with Targeted Drugs on Anticancer Treatment. Cancer Sci. 110 (1), 23–30. 10.1111/cas.13849 30358009 PMC6317954

[B8] Fleischmann A. Rotzer D. Seiler R. Studer U. E. Thalmann G. N. (2011). Her2 Amplification Is Significantly More Frequent in Lymph Node Metastases from Urothelial Bladder Cancer Than in the Primary Tumours. Eur. Urol. 60 (2), 350–357. 10.1016/j.eururo.2011.05.035 21640482

[B9] Galsky M. D. Hall S. J. (2010). Bladder Cancer: Current Management and Opportunities for a Personalized Approach. Mt Sinai J. Med. 77 (6), 587–596. 10.1002/msj.20224 21105122

[B10] Galsky M. D. Pal S. K. Lin S.-W. Ogale S. Zivkovic M. Simpson J. (2018). Real-World Effectiveness of Chemotherapy in Elderly Patients with Metastatic Bladder Cancer in the United States. Blc 4 (2), 227–238. 10.3233/blc-170149 PMC5929305 29732393

[B11] Grivas P. D. Day K. C. Karatsinides A. Paul A. Shakir N. Owainati I. (2013). Evaluation of the Antitumor Activity of Dacomitinib in Models of Human Bladder Cancer. Mol. Med. 19, 367–376. 10.2119/molmed.2013.00108 24166682 PMC3883970

[B12] Hayashi T. Seiler R. Oo H. Z. Jäger W. Moskalev I. Awrey S. (2015). Targeting HER2 with T-DM1, an Antibody Cytotoxic Drug Conjugate, Is Effective in HER2 over Expressing Bladder Cancer. J. Urol. 194 (4), 1120–1131. 10.1016/j.juro.2015.05.087 26047983

[B13] Hussain M. H. A. MacVicar G. R. Petrylak D. P. Dunn R. L. Vaishampayan U. Lara P. N. Jr. (2007). Trastuzumab, Paclitaxel, Carboplatin, and Gemcitabine in Advanced Human Epidermal Growth Factor Receptor-2/neu-Positive Urothelial Carcinoma: Results of a Multicenter Phase II National Cancer Institute Trial. Jco 25 (16), 2218–2224. 10.1200/jco.2006.08.0994 17538166

[B14] Jia X. Wang H. Li Z. Yan J. Guo Y. Zhao W. (2020). HER4 Promotes the Progression of Colorectal Cancer by Promoting Epithelial-mesenchymal T-ransition. Mol. Med. Rep. 21 (4), 1779–1788. 10.3892/mmr.2020.10974 32319604 PMC7057779

[B15] Jiang Q. Xie M.-X. Zhang X.-C. (2020). Complete Response to Trastuzumab and Chemotherapy in Recurrent Urothelial Bladder Carcinoma with HER2 Gene Amplification: A Case Report. Wjcc 8 (3), 594–599. 10.12998/wjcc.v8.i3.594 32110671 PMC7031824

[B16] Jin Q. Esteva F. J. (2008). Cross-talk between the ErbB/HER Family and the Type I Insulin-like Growth Factor Receptor Signaling Pathway in Breast Cancer. J. Mammary Gland Biol. Neoplasia 13 (4), 485–498. 10.1007/s10911-008-9107-3 19034632

[B17] Jorissen R. Walker F. Pouliot N. Garrett T. P. Ward C. W. Burgess A. W. (2003). Epidermal Growth Factor Receptor: Mechanisms of Activation and Signalling. Exp. Cel Res. 284 (1), 31–53. 10.1016/s0014-4827(02)00098-8 12648464

[B18] Juanpere N. Agell L. Lorenzo M. de Muga S. López-Vilaró L. Murillo R. (2012). Mutations in FGFR3 and PIK3CA, Singly or Combined with RAS and AKT1, Are Associated with AKT but Not with MAPK Pathway Activation in Urothelial Bladder Cancer. Hum. Pathol. 43 (10), 1573–1582. 10.1016/j.humpath.2011.10.026 22417847

[B19] Junttila T. T. Laato M. Vahlberg T. Söderström K. O. Visakorpi T. Isola J. (2003). Identification of Patients with Transitional Cell Carcinoma of the Bladder Overexpressing ErbB2, ErbB3, or Specific ErbB4 Isoforms: Real-Time Reverse Transcription-PCR Analysis in Estimation of ErbB Receptor Status from Cancer Patients. Clin. Cancer Res. 9 (14), 5346–5357. 14614020

[B20] Kim W. T. Kim J. Yan C. Jeong P. Choi S. Y. Lee O. J. (2014). S100A9 and EGFR Gene Signatures Predict Disease Progression in Muscle Invasive Bladder Cancer Patients after Chemotherapy. Ann. Oncol. 25 (5), 974–979. 10.1093/annonc/mdu037 24631944

[B21] Kiss B. Wyatt A. W. Douglas J. Skuginna V. Mo F. Anderson S. (2017). Her2 Alterations in Muscle-Invasive Bladder Cancer: Patient Selection beyond Protein Expression for Targeted Therapy. Sci. Rep. 7, 42713. 10.1038/srep42713 28205537 PMC5311866

[B22] Krüger S. Weitsch G. Büttner H. Matthiensen A. Böhmer T. Marquardt T. (2002). HER2 Overexpression in Muscle-Invasive Urothelial Carcinoma of the Bladder: Prognostic Implications. Int. J. Cancer 102 (5), 514–518. 10.1002/ijc.10731 12432555

[B23] Laé M. Couturier J. Oudard S. Radvanyi F. Beuzeboc P. Vieillefond A. (2010). Assessing HER2 Gene Amplification as a Potential Target for Therapy in Invasive Urothelial Bladder Cancer with a Standardized Methodology: Results in 1005 Patients. Ann. Oncol. 21 (4), 815–819. 10.1093/annonc/mdp488 19889613 PMC2844947

[B24] Lemmon M. A. Schlessinger J. (2010). Cell Signaling by Receptor Tyrosine Kinases. Cell 141 (7), 1117–1134. 10.1016/j.cell.2010.06.011 20602996 PMC2914105

[B25] Liukkonen T. J. O. Lipponen P. K. Helle M. Jauhiainen K. E. (1997). Immunoreactivity of Bcl-2, P53 and EGFr Is Associated with Tumor Stage, Grade and Cell Proliferation in Superficial Bladder Cancer. Urol. Res. 25 (1), 1–8. 10.1007/bf00941899 9079739

[B26] Liukkonen T. Rajala P. Raitanen M. Rintala E. Kaasinen E. Lipponen P. (1999). Prognostic Value of MIB-1 Score, P53, EGFr, Mitotic Index and Papillary Status in Primary Superficial (Stage pTa/T1) Bladder Cancer: A Prospective Comparative Study. Eur. Urol. 36 (5), 393–400. 10.1159/000020039 10516448

[B27] Machleidt A. Buchholz S. Diermeier-Daucher S. Zeman F. Ortmann O. Brockhoff G. (2013). The Prognostic Value of Her4 Receptor Isoform Expression in Triple-Negative and Her2 Positive Breast Cancer Patients. BMC Cancer 13, 437. 10.1186/1471-2407-13-437 24063248 PMC3849049

[B28] Memon A. A. Gilliver S. C. Borre M. Sundquist J. Sundquist K. Nexo E. (2018). Soluble HER3 Predicts Survival in Bladder Cancer Patients. Oncol. Lett. 15 (2), 1783–1788. 10.3892/ol.2017.7470 29434875 PMC5774496

[B29] Memon A. A. Sorensen B. S. Meldgaard P. Fokdal L. Thykjaer T. Nexo E. (2006). The Relation between Survival and Expression of HER1 and HER2 Depends on the Expression of HER3 and HER4: a Study in Bladder Cancer Patients. Br. J. Cancer 94 (11), 1703–1709. 10.1038/sj.bjc.6603154 16685269 PMC2361308

[B30] Memon A. A. Sorensen B. S. Melgard P. Fokdal L. Thykjaer T. Nexo E. (2004). Expression of HER3, HER4 and Their Ligand Heregulin-4 Is Associated with Better Survival in Bladder Cancer Patients. Br. J. Cancer 91 (12), 2034–2041. 10.1038/sj.bjc.6602251 15583696 PMC2409781

[B31] Modjtahedi H. Cho B. C. Michel M. C. Solca F. (2014). A Comprehensive Review of the Preclinical Efficacy Profile of the ErbB Family Blocker Afatinib in Cancer. Naunyn-schmiedeberg's Arch. Pharmacol. 387 (6), 505–521. 10.1007/s00210-014-0967-3 24643470 PMC4019832

[B32] Mollica V. Rizzo A. Montironi R. Cheng L. Giunchi F. Schiavina R. (2020). Current Strategies and Novel Therapeutic Approaches for Metastatic Urothelial Carcinoma. Cancers (Basel) 12 (6). 10.3390/cancers12061449 PMC7352972 32498352

[B33] Munk M. Memon A. Poulsen S. S. Borre M. Nexo E. Sorensen B. S. (2013). The HER4 Isoform JM-a/CYT2 Relates to Improved Survival in Bladder Cancer Patients but Only if the Estrogen Receptor α Is Not Expressed. Scand. J. Clin. Lab. Invest. 73 (6), 503–513. 10.3109/00365513.2013.818706 24015958

[B34] Normanno N. De Luca A. Bianco C. Strizzi L. Mancino M. Maiello M. R. (2006). Epidermal Growth Factor Receptor (EGFR) Signaling in Cancer. Gene 366 (1), 2–16. 10.1016/j.gene.2005.10.018 16377102

[B35] Oda K. Matsuoka Y. Funahashi A. Kitano H. (2005). A Comprehensive Pathway Map of Epidermal Growth Factor Receptor Signaling. Mol. Syst. Biol. 1, 2005. 10.1038/msb4100014 PMC1681468 16729045

[B36] Oudard S. Culine S. Vano Y. Goldwasser F. Théodore C. Nguyen T. (2015). Multicentre Randomised Phase II Trial of Gemcitabine+platinum, with or without Trastuzumab, in Advanced or Metastatic Urothelial Carcinoma Overexpressing Her2. Eur. J. Cancer 51 (1), 45–54. 10.1016/j.ejca.2014.10.009 25459391

[B37] Powles T. Huddart R. A. Elliott T. Sarker S.-J. Ackerman C. Jones R. (2017). Phase III, Double-Blind, Randomized Trial that Compared Maintenance Lapatinib versus Placebo after First-Line Chemotherapy in Patients with Human Epidermal Growth Factor Receptor 1/2-Positive Metastatic Bladder Cancer. Jco 35 (1), 48–55. 10.1200/jco.2015.66.3468 28034079

[B38] Pruthi R. S. Nielsen M. Heathcote S. Wallen E. M. Rathmell W. K. Godley P. (2010). A Phase II Trial of Neoadjuvant Erlotinib in Patients with Muscle-Invasive Bladder Cancer Undergoing Radical Cystectomy: Clinical and Pathological Results. BJU Int. 106 (3), 349–354. 10.1111/j.1464-410x.2009.09101.x 20089114

[B39] Rose M. Maurer A. Wirtz J. Bleilevens A. Waldmann T. Wenz M. (2020). EGFR Activity Addiction Facilitates Anti-ERBB Based Combination Treatment of Squamous Bladder Cancer. Oncogene 39 (44), 6856–6870. 10.1038/s41388-020-01465-y 32978523 PMC7605436

[B40] Roskoski R. Jr. (2014). The ErbB/HER Family of Protein-Tyrosine Kinases and Cancer. Pharmacol. Res. 79, 34–74. 10.1016/j.phrs.2013.11.002 24269963

[B41] Sarfaty M. Rosenberg J. E. (2020). Antibody-Drug Conjugates in Urothelial Carcinomas. Curr. Oncol. Rep. 22 (2), 13. 10.1007/s11912-020-0879-y 32008109

[B42] Sheng X. Yan X. Wang L. Shi Y. Yao X. Luo H. (2021). Open-label, Multicenter, Phase II Study of RC48-ADC, a HER2-Targeting Antibody-Drug Conjugate, in Patients with Locally Advanced or Metastatic Urothelial Carcinoma. Clin. Cancer Res. 27 (1), 43–51. 10.1158/1078-0432.ccr-20-2488 33109737

[B43] Slojewski M. (2000). Results of Radical Cystectomy for Management of Invasive Bladder Cancer with Special Reference to Prognostic Factors and Quality of Life Depending on the Type of Urinary Diversion. Ann. Acad. Med. Stetin 46, 217–229. 11712306

[B44] Su W.-P. Tu I.-H. Hu S.-W. Yeh H.-H. Shieh D.-B. Chen T.-Y. (2007). HER-2/neu Raises SHP-2, Stops IFN-γ Anti-proliferation in Bladder Cancer. Biochem. Biophysical Res. Commun. 356 (1), 181–186. 10.1016/j.bbrc.2007.02.099 17346677

[B45] Sung H. Ferlay J. Siegel R. L. Laversanne M. Soerjomataram I. Jemal A. (2021). Global Cancer Statistics 2020: GLOBOCAN Estimates of Incidence and Mortality Worldwide for 36 Cancers in 185 Countries. CA A. Cancer J. Clin. 71 (3), 209–249. 10.3322/caac.21660 33538338

[B46] Tsai Y.-C. Ho P.-Y. Tzen K.-Y. Tuan T.-F. Liu W.-L. Cheng A.-L. (2015). Synergistic Blockade of EGFR and HER2 by New-Generation EGFR Tyrosine Kinase Inhibitor Enhances Radiation Effect in Bladder Cancer Cells. Mol. Cancer Ther. 14 (3), 810–820. 10.1158/1535-7163.mct-13-0951 25589492

[B47] Wang A. Jiang A. Gan X. Wang Z. Huang J. Dong K. (2020). EGFR-AS1 Promotes Bladder Cancer Progression by Upregulating EGFR. Biomed. Res. Int. 2020, 6665974. 10.1155/2020/6665974 33426060 PMC7781701

[B48] Wezel F. Erben P. Gaiser T. Budjan J. von Hardenberg J. Michel M. S. (2018). Complete and Durable Remission of Human Epidermal Growth Factor Receptor 2-Positive Metastatic Urothelial Carcinoma Following Third-Line Treatment with Trastuzumab and Gemcitabine. Urol. Int. 100 (1), 122–125. 10.1159/000443280 26780095

[B49] Wong Y.-N. Litwin S. Vaughn D. Cohen S. Plimack E. R. Lee J. (2012). Phase II Trial of Cetuximab with or without Paclitaxel in Patients with Advanced Urothelial Tract Carcinoma. Jco 30 (28), 3545–3551. 10.1200/jco.2012.41.9572 PMC3454772 22927525

[B50] Wülfing C. Machiels J.-P. H. Richel D. J. Grimm M.-O. Treiber U. De Groot M. R. (2009). A Single-Arm, Multicenter, Open-Label Phase 2 Study of Lapatinib as the Second-Line Treatment of Patients with Locally Advanced or Metastatic Transitional Cell Carcinoma. Cancer 115 (13), 2881–2890. 10.1002/cncr.24337 19399906

[B51] Yan M. Schwaederle M. Arguello D. Millis S. Z. Gatalica Z. Kurzrock R. (2015). HER2 Expression Status in Diverse Cancers: Review of Results from 37,992 Patients. Cancer Metastasis Rev. 34 (1), 157–164. 10.1007/s10555-015-9552-6 25712293 PMC4368842

[B52] Yarden Y. Sliwkowski M. X. (2001). Untangling the ErbB Signalling Network. Nat. Rev. Mol. Cel Biol 2 (2), 127–137. 10.1038/35052073 11252954

[B53] Yun S. Koh J. Nam S. K. Park J. O. Lee S. M. Lee K. (2018). Clinical Significance of Overexpression of NRG1 and its Receptors, HER3 and HER4, in Gastric Cancer Patients. Gastric Cancer 21 (2), 225–236. 10.1007/s10120-017-0732-7 28573357

